# Knockout of the ING5 epigenetic regulator confirms roles in stem cell maintenance and tumor suppression *in vivo*

**DOI:** 10.1371/journal.pone.0313255

**Published:** 2025-01-09

**Authors:** Buthaina Al Shueili, Arthur Dantas, Etienne Mahe, Tak Ho Chu, Yang Yang, Elodie Labit, Eren Kutluberk, Nicolas Lasaleta, Anand Masson, Hiba Omairi, Kenichi Ito, Roman J. Krawetz, Rajiv Midha, Gregory Cairncross, Karl Riabowol

**Affiliations:** 1 Robson DNA Sciences Centre, Calgary, Canada; 2 Arnie Charbonneau Cancer Institute, Calgary, Canada; 3 Departments of Biochemistry & Molecular Biology, University of Calgary, Calgary, Canada; 4 Departments of Pathology and Laboratory Medicine, Cumming School of Medicine, University of Calgary, Calgary, Alberta, Canada; 5 Hotchkiss Brain Institute, University of Calgary, Calgary, Canada; 6 Departments of Clinical Neurosciences, University of Calgary, Calgary, Canada; 7 Departments of Alberta Children’s Hospital Research Institute, University of Calgary, Calgary, Canada; 8 Departments of Comparative Biology and Experimental Medicine, University of Calgary, Calgary, Canada; 9 Departments of McCaig Institute for Bone & Joint Health, University of Calgary, Calgary, Canada; 10 Departments of Cell Biology and Anatomy, University of Calgary, Calgary, Canada; 11 Departments of Surgery, University of Calgary, Calgary, Canada; 12 Departments of Oncology, University of Calgary, Calgary, Canada; Fujita Health University, JAPAN

## Abstract

INhibitor of Growth (ING1-5) proteins are epigenetic readers that target histone acetyltransferase (HAT) or histone deacetylase (HDAC) complexes to the H3K4Me3 mark of active transcription. ING5 targets Moz/Morf and HBO1 HAT complexes that alter acetylation of H3 and H4 core histones, affecting gene expression. Previous experiments *in vitro* indicated that ING5 functions to maintain stem cell character in normal and in cancer stem cells. Here we find that CRISPR/Cas9 ING5 knockout (KO) mice are sub-fertile but show no decrease in lifespan or ability to heal wounds despite indications of depleted stem cell pools in several tissues. ING5 KO mouse embryo fibroblasts accumulate in G2 of the cell cycle, have high levels of abnormal nuclei and show high basal levels of the γH2AX indicator of DNA damage. KO animals also develop severe dermatitis at a 5-fold higher rate that wild-type littermates. Consistent with ING5 serving a tumor suppressive role, ING5 KO mice developed germinal centre diffuse large B-cell lymphomas at a rate 6-fold higher than control mice at 18 months of age. These data suggest that ING5 functions *in vivo* to maintain stem cell character in multiple organs, that reduction of stem cell populations is not limiting for murine lifespan and that like a subset of other ING family members, ING5 functions as a tumor suppressor in hematopoietic cells *in vivo*.

## Introduction

Stem cells are defined by two fundamental characteristics, self-renewal and multipotency [1]. The maintenance of stem cell character was widely attributed to transcription factors that regulate stem cell behaviour in a dynamic manner [2]. However, it has become evident that alteration in chromatin accessibility by epigenetic factors also governs the ability of transcription factors to exert their effects during development [3]. Embryonic stem cells (ESCs), for instance, have a distinct transcriptional profile accompanied by a unique epigenetic landscape, and as they proceed with differentiation, genes that are needed for pluripotency are silenced by histone modifications and/or DNA methylation, and hence these cells accumulate epigenetic markers that differentiate them from pluripotent cells [4]. Histone post-translational modification (PTM) occurs by many mechanisms, most commonly on lysine residues (methylation, acetylation, biotinylation, ubiquitylation, sumoylation, ADP-ribosylation and others) and phosphorylatable amino acids [5,6] to regulate nucleosome density and therefore chromatin accessibility to transcription factors. In general, histone methylation has been mostly linked to gene repression, while histone acetylation strongly correlates with transcriptional activation [7], however this simple model has been expanded to include the function of bivalent chromatin in which both negative and positively acting epigenetic marks are found on genes important in mammalian development [8]. Lysine acetylation is particularly connected with cell metabolism and states of differentiation since it is very sensitive to the concentrations of its cofactors NAD+, acetyl-CoA and acyl-CoA [9].

Epigenetic regulators have been characterized as *writers* that deposit the modification, *erasers* that remove them and *readers* that recognize these modifications [5,6]. The INhibitor of Growth (ING1-5) family fall into the reader category since they specifically recognize the H3K4Me3 epigenetic mark with their conserved plant homeodomain (PHD) form of zinc finger in a methylation-sensitive manner [10]. They are stoichiometric components of histone acetyltransferase (HAT) or histone deacetylase (HDAC) complexes [11,12], and they target these complexes to affect local acetylation levels. The first member of the ING family was discovered by our group using an *in vivo* tumor suppressor screen as a gene downregulated in breast cancer [13] and ING family members have subsequently been implicated in regulating proliferation and growth [14], DNA damage repair [15,16], senescence [17], and stem cell regeneration [18,19].

ING5 shows 67% amino acid identity with ING4 [20], and distinct from other ING family proteins, it is a stoichiometric member of two distinct HAT complexes. Unlike ING4 which was found primarily in the HBO1 complex, ING5 was found in both HBO1 and Moz/Morf HATs [12], and so depending on its catalytic acetyltransferase subunit it can target either histone H3 or H4 suggesting a unique role for ING5 as a regulator of HAT affinity to different histone tails. Like other INGs, ING5 targeting HAT complexes allows it to regulate cell growth in different pathways. In lung cancer, ING5 overexpression inhibits the epithelial-to-mesenchymal transition (EMT) by promoting phosphorylation‐dependent degradation of β‐catenin, leading to downregulation of WNT/β‐catenin signaling [21]. A strong interaction of ING5 with p53, specifically via regulating p53 acetylation status has been reported to activate the p53 pathway, inhibit proliferation, and enhance DNA repair [15,22]. A role for ING5 in differentiation was also highlighted in results from an unbiased siRNA-based genome-wide screen for regulators of epidermal stem cell character [23]. ING5 was one of five epigenetic regulators identified to play strong roles in maintaining epidermal stem cell renewal and prevented cells from differentiating by functionally interacting with ZMAT2, which regulates the splicing of genes differentially expressed during differentiation [24]. A similar observation was noted by our group when studying stem-like brain tumor initiating cells (BTICs). ING5 was not only highly expressed in these cancer stem cells and lost upon differentiation, but it also promoted self-renewal, prevented lineage differentiation, and increased stem cell pools [18]. Consistent with these observations, a subsequent study using a Set1A synthetic lethality screen, showed that ING5 and the Set1A complex are major players in maintaining ESC self-renewal and regulating differentiation [19]. Examination of the livers and spleens of ING5 KO mice indicated a loss of hematopoietic stem cells and hypoplastic spleens [25], cardiac ventricular septal defects [26] and similar to ING3 knockout [27], simultaneous loss of the related ING4 and ING5 proteins results in arrested development and embryonic lethality [26]. In addition to the synthetic lethality seen between ING5 and Set1A, ING5 was also reported to be synergistic lethal with other members of the HBO1 (KAT7) acetylation complex [28].

Aside from effects on stem cell differentiation [18,23,25,26], it is unknown what the different functions of ING5 might be in normal animal development, but single knockouts of other ING family members suggest the effects may be profound [29]. Knockout of ING1 resulted in smaller mice developing enlarged spleens, a higher level of apoptosis, increased formation of B-cell lymphomas [30], and hypersensitivity to radiation [31]. Male ING2 deficient mice were infertile due to spermatogenesis defects where sperm counts were only 2% of normal and they developed histiocytic sarcomas [32]. Mice lacking ING3 did not survive past E10.5, with embryos displaying severe growth retardation and a failure of neural tube closure [27]. Mice lacking ING4 developed normally but were hypersensitive to lipopolysacharride (LPS) treatment, and knockouts exhibited high morbidity due to the activation of the NF-κB pathway and subsequent overproduction of various cytokines [33].

In this study we address the phenotypic effects of ING5 knockout by generating a CRISPR/Cas9 knockout model and asking whether the role(s) of ING5 *in vitro* in maintaining stem cell character are maintained *in vivo*. We find that although viability and lifespan were unaffected through four generations of mice, knockouts were less fertile, showed an altered sex ratio, increased thinning of the dermis with age and a cell cycle defect in mitosis. KO animals also expressed more differentiated brain progenitors, produced fewer hair follicles containing stem cells after skin wounding, had slower cartilage wound closure, and displayed major changes in fibroblast morphology consistent with the loss of ING5 promoting premature differentiation of stem cells.

## Material and methods

### Generation of ING5 CRISPR/Cas9 knockout mice and genotyping

ING5^-/-^ mice were generated in the Centre for Genome Engineering at the University of Calgary using CRISPR/Cas9 and used for experiments through 4 generations of breeding. Briefly, three independent guide RNAs were designed to target exon3 that recognize regions that are unique between ING5 and the related ING4 gene. Cas9 mRNA and sgRNAs were microinjected into fertilized embryos of C57BL/6J mice. Mutations in ING5 were confirmed by sequencing analyses. Multiple mutations were generated, and two mutations (1bp and 8 bp deletions) were kept for heterozygote crossing. Homozygous KO mice were born from heterozygous intercrosses. All analyses were done in parallel with age matched wild-type (WT) littermates as a control group. All mice were genotyped after weaning 20 days after birth, using AccuStart™ II Mouse Genotyping Kits (Cat. No. 95135–500) with specific primers.

Primers for confirming the sequence of putative KO animals were:

ING5F (1bp): CATATGGGTGTGCTGCTGTC

ING5R (1bp): CACTTGCTTGCACACTCTCC

ING5F (8bp): CCTCTCTCCCTGAAACCACA

ING5R (8bp): CAGATGAAGACCCCCAGTGT

### RNA isolation and Real-Time quantitative PCR (RT-qPCR)

Tissues were preserved in RNAlater (cat no. AM7023) prior to extraction. For brain analyses, pieces of brain tissue adjacent to the subventricular zone were used for RNA isolation. The exact weight of the brain tissue used for RNA isolation was as suggested by instructions for Quiagen RNeasy Mini Kits (cat no. 74134). RNA isolation was followed by reverse transcription using an ABI High-Capacity cDNA synthesis kit according to the manufacturer’s instructions. cDNA samples were amplified using SYBER Green master mix and using an ABI 7500 Fast qPCR machine. The following primers were used, and relative fold change was calculated using ΔΔCT relative to the 18S housekeeping gene.

ING5F1: GGACAGCATTGAGAACCTTCCC

ING5R1: GGGCTGACGAGAGAGTCTTCACTG

ING5F2: CCATGCAGACCTACGAGATGGTGG

ING5R2: GACTCCGGCCTTTTTTCA AGCTCCG

18SF: ACTGCCATTAAGGGCGTGGG

18SR: GCCCTCTTGGTGAGGTCGAT

Sox2F: CACATGAAGGAGCACCCGGA

Sox2R: CCTCCGGGAAGCGTGTACTT

NestinF: CGACAACCTTGCCGAAGAGC

NestinR: CAGAGCCTCTAACTCGCGCT

GFAPF: CAGGAAATTGCTGGAGGGCG

GFAPR: CCAGGCTGGTTTCTCGGATCT

### Mouse embryo fibroblast isolation

13.5-day embryos were isolated, minced and digested after mincing with 2mg/mL collagenase (cat no. LS004176). Digested cells were plated in 60mm plates with Dulbecco’s Modified Eagle’s Medium, 10% fetal bovine serum, and 1X penicillin and streptomycin. Attached fibroblasts were passaged once when reaching 80% confluency, trypsinized with 0.25% trypsin/0.1% ethylenediaminetetraacetic acid (trypsin/EDTA) solution and were then frozen and stored in liquid N_2_. Upon thawing, cells were expanded and incubated in 5% oxygen and 5% CO_2_ in humidified incubators to prevent premature senescence from oxidative stress [34].

### Immunohistochemistry and Immunocytochemistry

Tissues were fixed in 4% paraformaldehyde (PFA) overnight followed by paraffin embedding. For antigen retrieval, sections (5μm thick) were heated in a microwave oven for 2 min at 100% power, followed by 20 min at 20% power using Rodent Decloaker AR buffer (Biocare Medical). Samples were then treated with Peroxidase Block and incubated for 15 min with 1% BSA in TBST wash buffer at room temperature. Samples were then incubated with a 1:200 dilution of primary antibody (anti-Doublecortin (DCX) ab18723) overnight at 4°C. Analysis of DCX expression was made by Labeled Polymer-HRP anti-mouse reagent for 1h at RT and a 5 min incubation with DAB+ substrate and chromogen (DAKO, K3467). Lastly, slides were counterstained with Mayer’s hematoxylin (DAKO, S3309). For staining with the thymidine analog ethynyl deoxyuridine (EdU), cells were fixed with 4% PFA, washed with PBS buffer, and stained for EdU following the instructions in the Click-iT™ EdU Imaging Kit (cat no C10086). Antibodies used for staining tumor samples were rat anti-CD3 (1:100, ab 11089), rabbit anti-CD10 (1:200, ab 256494), rabbit anti-CD20 (1:100, ab 64088), rabbit anti-CD34 (1:500, ab 316277), rabbit anti-terminal deoxynucleotidyl transferase (1:250, ab76544) with cells counterstained with hematoxylin to visualize nuclei. For DCX staining, cells were mounted using Antifade Mounting Medium with DAPI (cat no. H-1500), and images were taken using a Zeiss LSM 880 Confocal Microscope.

For nerve staining, the nerves were washed in phosphate-buffered saline and placed in sucrose for 2 weeks at 4°C. Nerves were then embedded in optimal cutting temperature compound and sectioned on a cryostat at a thickness of 10μm longitudinally. Sections were stained with neurofilament heavy chain (NF-H, 1:1000, Biolegend), p75 (Biolegend, 1:500), and MPZ (Biolegend, 1:500) antibodies. Finally, sections were counterstained with DAPI for nuclear staining (1:2000) for 5 min and slides were covered with fluorescent mounting medium (FluorSave Reagent, Millipore). Sections were examined in a blinded fashion, initially with a fluorescence microscope (Olympus BX51; Olympus, Japan) and imaged with a slide scanner (Olympus VS110-S5 Slidescanner, Japan).

### Flow cytometry

1x10^5^ WT or KO MEFs were seeded in 60 mm cell culture dishes for cell cycle analysis. Twenty-four hours after seeding, DMEM+10% FBS media was removed, and DMEM without growth factors was added to the cells for synchronization by incubation under low (LO; 5%) oxygen for 48 hours. DMEM+10% FBS was added back to the cells, and the plates were divided into groups for LO and HO incubators. In each of the next six days, cells were trypsinized, washed with PBS once and resuspended in 1 ml of 0.9% NaCl, following which, samples were vortexed gently while slowly adding 1 ml of ice-cold 95% ethanol drop by drop. Samples were incubated at room temperature for 30 minutes, then transferred to 4°C and stored until all other time points were collected. Then, cells were centrifuged, and the fixing solution was removed. Lastly, cells were resuspended in 1 ml 5 μg/ml of propidium iodide solution and incubated for 30 mins at room temperature prior to cell cycle analysis at The Flow Cytometry Facility at the University of Calgary. Means between samples were compared using a two-tailed t-test and standard error; the p-value was considered significant if <0.05.

### Examination of nuclei

2x10^4^ MEFs were seeded per 22x22mm coverslip (VWR Canada) and cultured in DMEM+10% FBS in LO and HO conditions for five days. Each day, cells were fixed with 3.2% paraformaldehyde plus 0.25% glutaraldehyde in PEM cytoskeletal buffer (0.1M PIPES, 0.001M EDTA, 0.001M MgCl_2_, 0.5% v/v Triton-X-100). Immunofluorescence staining was performed using alpha-tubulin primary antibody (Novus, DM1A, 1:10000) and Hoechst 33342 (1:10000) (Thermo-Fisher Scientific) to stain DNA. Images (Z-stacks) were acquired with a ZEISS LSM880 confocal microscope and images for quantification were acquired with a Zeiss AxioObserver Z1 epifluorescence microscope. Image analysis was performed using FIJI/Image J. The frequency of nuclear abnormalities in MEFs under different growth conditions was determined by counting in a blind experimental protocol. A total of 120 cells were counted per condition per time point. Means of nuclear abnormalities between samples were compared using a two-tailed t-test and standard error; the p-value was considered significant if <0.05.

### γH2AX staining

For DNA double-strand break assessment, 1x10^5^ WT or KO MEFs were seeded on 22x22mm coverslips (VWR Canada) and cultured in DMEM+10% FBS in LO conditions. Cells were then fixed as described above and stained for ɣH2AX (ab26350, ABCAM) and 53BP1 (ab21083, ABCAM). Secondary antibodies used for image acquisition were Alexa Fluor® 488-conjugated goat anti-mouse IgG (ab150113) or Alexa Fluor® 647-conjugated goat anti-rabbit IgG preadsorbed against human, mouse and rat immunosorbents (ab150083). Images were acquired using a Zeiss AxioObserver Z1 Epifluorescence Microscope.

### Ear punch wound healing models

For ear punches, eight-week-old mice were anaesthetized using isoflurane anaesthesia (5% induction, 2% maintenance). 2- and 4-mm punches were given to the centre of the right and left ear, respectively. The closure of the wound was monitored every week for 4 weeks with images taken with size standard and quantification of the wound area was performed using ImageJ.

### Wound-Induced Hair follicle Neogenesis (WIHN) assay

Full-thickness excision wounds were performed on mice pretreated with the analgesic meloxicam (Metacam®, 25 mg/kg reconstituted in 100 μL of sterile saline) subcutaneously and anesthetized using isofluorane (5% induction; 3% maintenance). Full-thickness square excisions (≥1.5cm diameter) were made on the mid-dorsal skin and analgesia (25 mg/kg Metacam® reconstituted in 100 μL of sterile saline) was provided subcutaneously 24 h post-wounding. Wounds closure was monitored every week, and healed skin was harvested 4 weeks post-wound. Neogenic hair follicles (HFs) were quantified by whole-mount imaging using a V5 Slide Scanner (Olympus Life Science).

### Peripheral Nerve Injury model

The details of the nerve crush injury are as described previously [35]. Briefly, procedures were performed on hindlimbs, following shaving, along with 70% ethanol aseptic preparation. The sciatic nerve was exposed and crushed using fine flat #5 jewelers’ forceps applying a continuous force. The opposite leg served as an uninjured control and the crush site was verified visually followed by irrigation with normal saline. The skin incision was then closed with single horizontal 6–0 Prolene sutures. 4 weeks post injury, the sciatic nerves were isolated and stored in 4% paraformaldehyde overnight for further staining. For axon analysis and thickness determination, animals were euthanized at 4 weeks after injury. Segments of distal nerve were harvested and fixed in 2.5% glutaraldehyde overnight at 4°C. The specimens were post-fixed in 2% osmium tetroxide followed by dehydration with graded acetone and then embedded in Epon resin. The sections were cut to 1 μm thickness and stained with 0.5% Toluidine Blue O. For histomorphometric analyses, whole nerve semithin sections were scanned with a slide scanner (VS110, Olympus, Japan) at 100x. Images were analyzed using SEM prediction model in AxonDeepSeg [36].

### Cytokine and blood analysis

Peripheral blood was isolated from mice using cardiac puncture. Mice were anesthetized with 5% isofluorane, 1ml of blood was collected using a 25G syringe punctured directly and slowly through the heart. For Complete Blood Count (CBC) analysis, the samples were collected in ETDA tubes to prevent clotting, and analyses were conducted by IDEXX Reference Laboratories. For cytokine analysis, samples were allowed to clot for 15min followed by centrifugation at 4°C for 10min to collect the serum supernatant. Samples were then analyzed using a 32-plex mouse cytokine panel by Eve Technologies.

### Statistics

At least 3 biological replicates were used for each test listed. All results are given as means ± SEM. For significance between two groups, comparisons were made using unpaired 2-tailed t-tests. All statistical analyses were carried out using GraphPad Prism 7.0 software and a two-tailed P value with 95% confidence interval was acquired. Values of p < 0.05 were considered significant.

## Results

### Generation of ING5 knockout mice using CRISPR/Cas9

**Fig 1:**
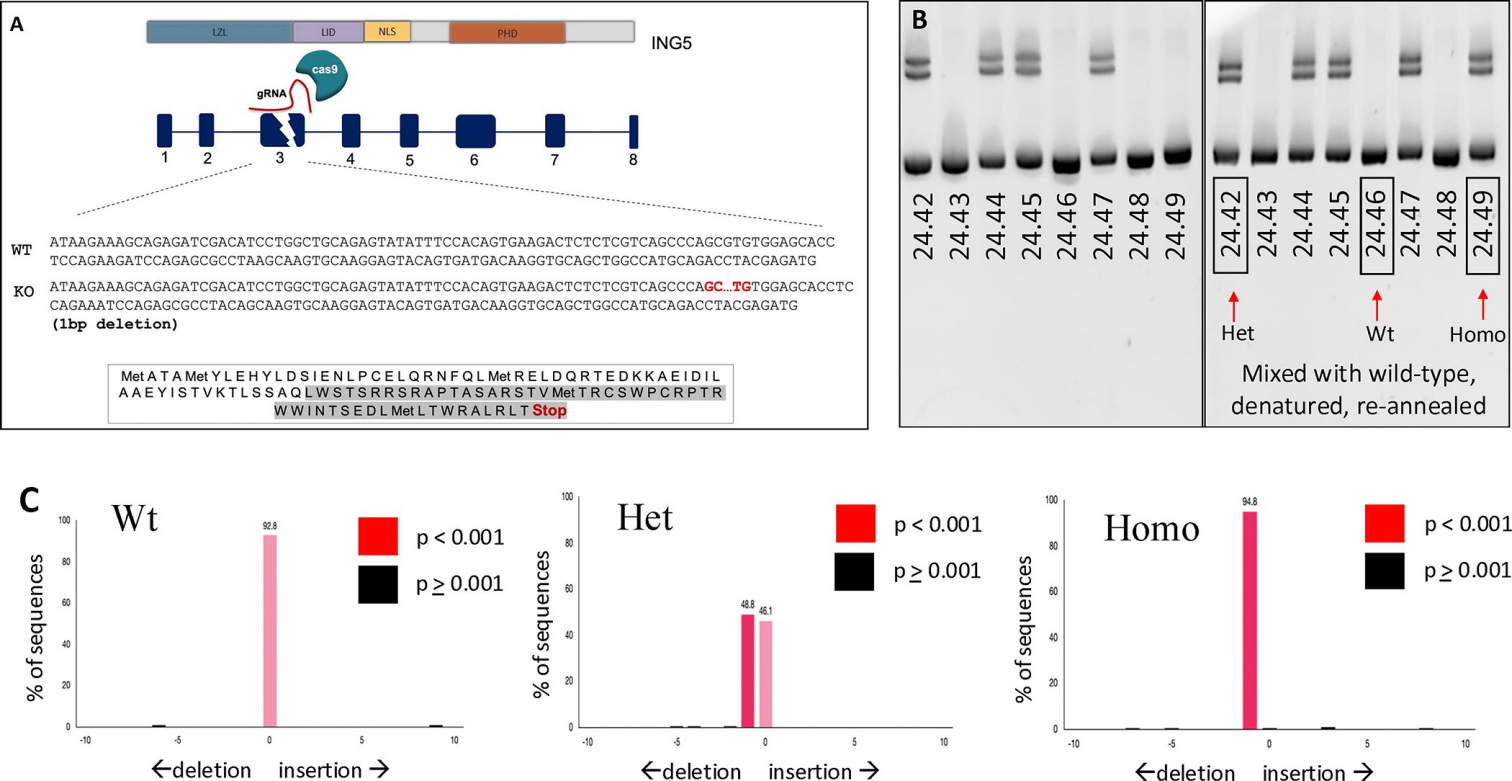
Generation of CRISPR/Cas9 ING5 knockout (KO) mice. **A)** The CRISPR/Cas9 targeting strategy used for generating ING5 knockout (KO) mice. Exons are indicated in blue boxes, and introns are noted by black lines. The targeting site for each of the guide RNAs (gRNAs) used is in exon3 (a unique site that distinguishes ING5 from ING4). The nucleotide and amino acid (in black) sequences of exon3 in wild-type (WT) and KO alleles are shown. The Gray area represents exon3 amino acids with a premature stop codon. **B)** For genotyping line 24 (8bp deletion), DNA was isolated from mouse ear samples and amplified using ING5 primers. PCR products analyzed on 10% polyacrylamide gels showing a single band are wild-type while heterozygous samples give additional retarded band(s). For homozygous KO samples, wildtype DNA is mixed with the samples, denatured, and re-annealed. If the sample is homozygous mutant, two bands are observed. **C)** For genotyping of the one bp deletion line, DNA was isolated from mouse ear samples, amplified using ING5 primers and PCR products were purified for sequencing and then analyzed using TIDE. Wild-type samples show bars with zero mutation, heterozygous samples are identified with two bars; one with zero mutation and the other with a 1bp deletion and homozygous samples give one bar with a 1bp deletion.

To generate ING5 knockouts, three different guide RNAs were microinjected into zygotes by personnel at Transgenic Services, Clara Christie Centre for Mouse Genomics (CCCMG) at the University of Calgary. For heterozygote crossing, two of these lines were chosen to produce a homozygote mouse. Line 2 produced a one base pair deletion, and line 24 produced an 8bp deletion, both of which produced frame shifts and subsequent premature stop codons (Fig 1A). Genotyping was carried out using PCR with ING5-specific primers and products were analyzed in 10% polyacrylamide gels (PAGE) to visualize differences of a few base pairs. For line 24 (8bp deletion), both wild-type and homozygous KO samples yielded non-retarded homoduplex bands which look identical on gels. In contrast, heterozygotes showed two additional bands of lower mobility. To determine if a sample was homozygous, the samples were mixed with wild-type DNA then heated and re-annealed. If the sample is from a homozygous mutant, additional bands with retarded mobility were seen (Fig 1B). However, for lines with 1bp deletions which cannot be resolved by PAGE, samples were sequenced and analyzed for mutation using online TIDE analysis tool (https://tide.deskgen.com) (Fig 1C). Analysis of ING5 transcription in different tissues was carried out for two distinct sequences, one upstream and one downstream of the mutation site to assay for potential truncated expression or transcriptional re-initiation. Loss of ING5 mRNA expression in multiple tissues from mice with two independent mutations confirmed ING5 was knocked out (S1 Fig).

### ING5 knockout progeny and demography

**Fig 2:**
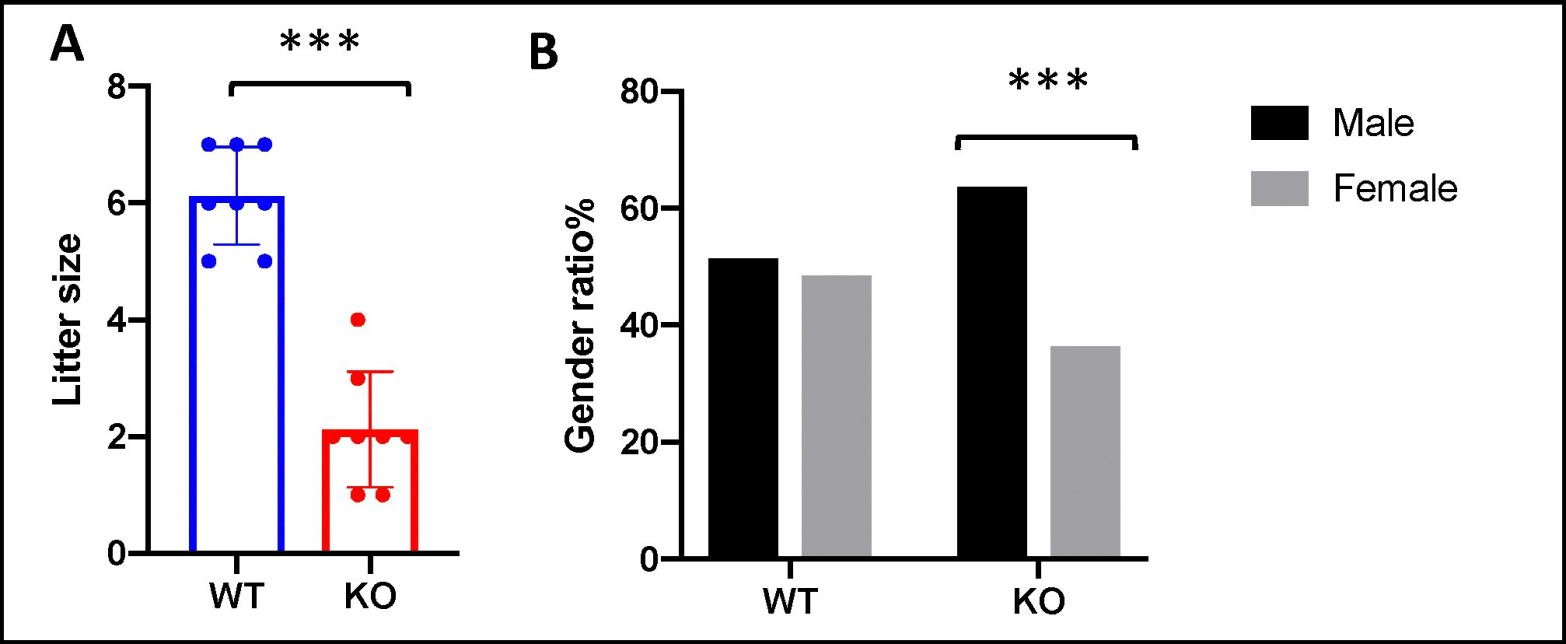
ING5 knockout progeny and demography. **A)** Litter sizes from WT and KO matings. Individual data points represent 1 mating that resulted in a litter. Graph shows mean ±SEM, ***p<0.0005. **B)** Percentage of males versus females in WT and KO mice. Total numbers of WT animals examined was 192 males and 181 females and in KO animals 128 males and 73 females. ***p<0.005.

To examine the basic physiology of ING5^-/-^ mice, they were monitored over four generations for body weight, lifespan, litter size and sex ratio. The knockouts initially were viable, alert and responsive with no physical complications obvious. The percentage of homozygous pups was approximately 12%, significantly less than expected from a normal Mendelian distribution of 25%, suggesting a level of early embryonic lethality. Related to this, the litter size of ING5 knockout mice is also very small compared to their wildtype litter mates (2 pups vs. 6 pups, respectively) (Fig 2A). Average weight was similar in WT and KO animals. With ageing, a significantly greater proportion of KOs developed severe dermatitis and B-cell lymphoma (described below), and although this necessitated euthanasia, this did not appear to significantly affect the overall lifespan of unaffected KO animals. The ratio of males:females in knockout animals deviated towards males with a 64:36% male:female ratio in the KO (n = 201) compared to a 51:49% male:female ratio in WT mice (n = 373) (Fig 2B).

### Reduction in neural stem cell markers and an increase in differentiated progenitors in brains of knockout mice

**Fig 3:**
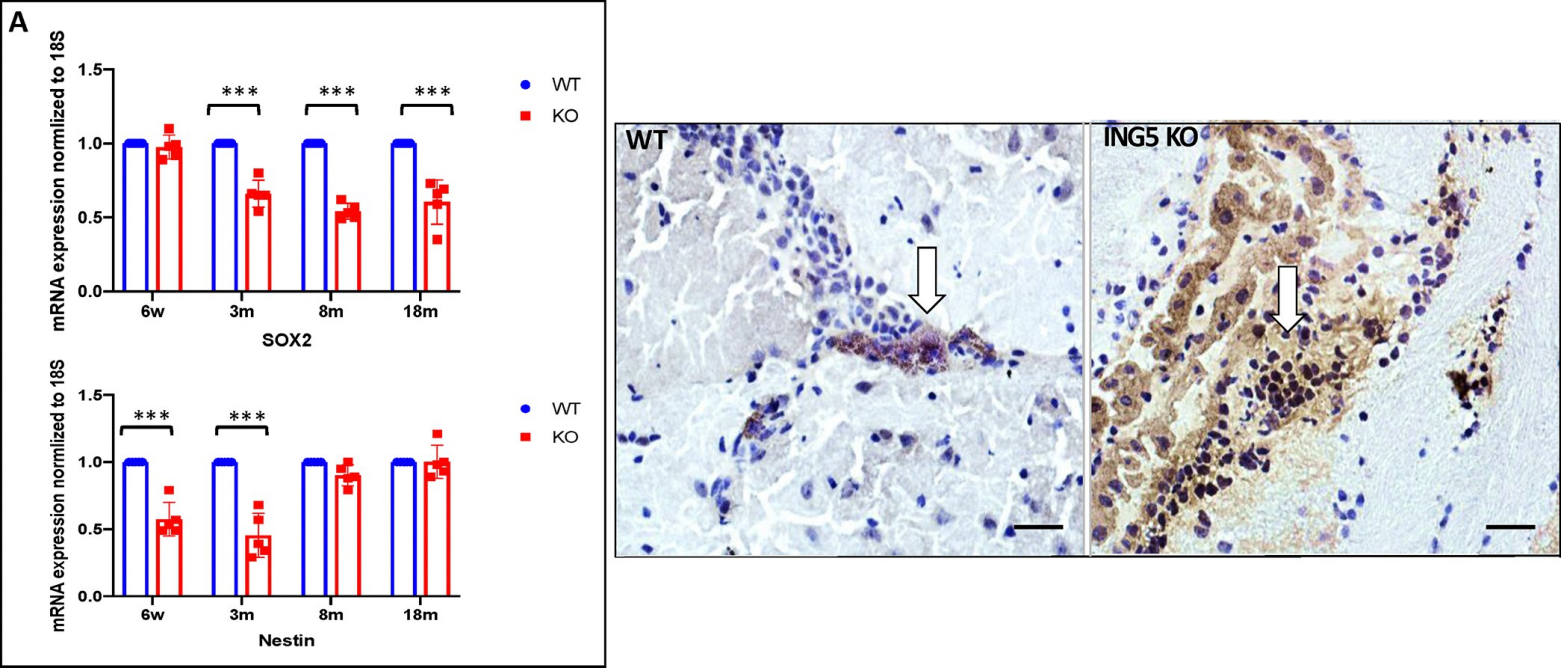
ING5 KO mice show reduction in neural stem cell markers and an increase in differentiated progenitors in brain. **A)** mRNA levels of the SOX2 and nestin neural stem cell markers in ING5 KO and WT brain tissues at 1.5, 3, 8 and 18 months. The graph shows mean ±SEM. (n = 5), ***p<0.005. **B)** Indirect immunostaining of 3-month-old WT and KO mice for the differentiation marker doublecortin (DC) in the brain subventricular zone. Arrows identify cells with brown positive staining and nuclei staining blue. The percentage of positive cells in WT in 4 fields was 8.8 ± 1% and in the KO 31.3 ± 24% (n = 3). DC expression is a measure of neural stem cells differentiating into neural precursor cells (NPCs) and forming newly differentiated neurons. Scale bars = 50mM.

We previously found that ectopic expression of ING5 increased the expression of the Oct4, Olig2 and nestin neural stem cell markers in stem-like brain tumour initiating cells (BTICS) *in vitro* while promoting self-renewal, preventing lineage differentiation, and increasing stem cell pools [18]. Examination of samples from the brain subventricular zone of KO mice showed that loss of ING5 resulted in decreased expression of the Sox2 and nestin stem cell markers at different ages with nestin being reduced by >50% 3 months after birth and Sox2 progressively decreasing throughout the lifespan (Fig 3A). Examination of the subventricular zone of the brain for doublecortin, a microtubule-associated protein expressed by newly differentiated migrating neurons confirmed that >3-fold more cells in the ING5 KO animals expressed doublecortin (Fig 3B) consistent with ING5 loss promoting the differentiation and growth of neural stem cells into neurons *in vivo*, similar to observations previously reported using *in vitro* cell models [18].

### ING5 KO mice produce fewer hair follicles after wounding and show slower ear wound closure

**Fig 4:**
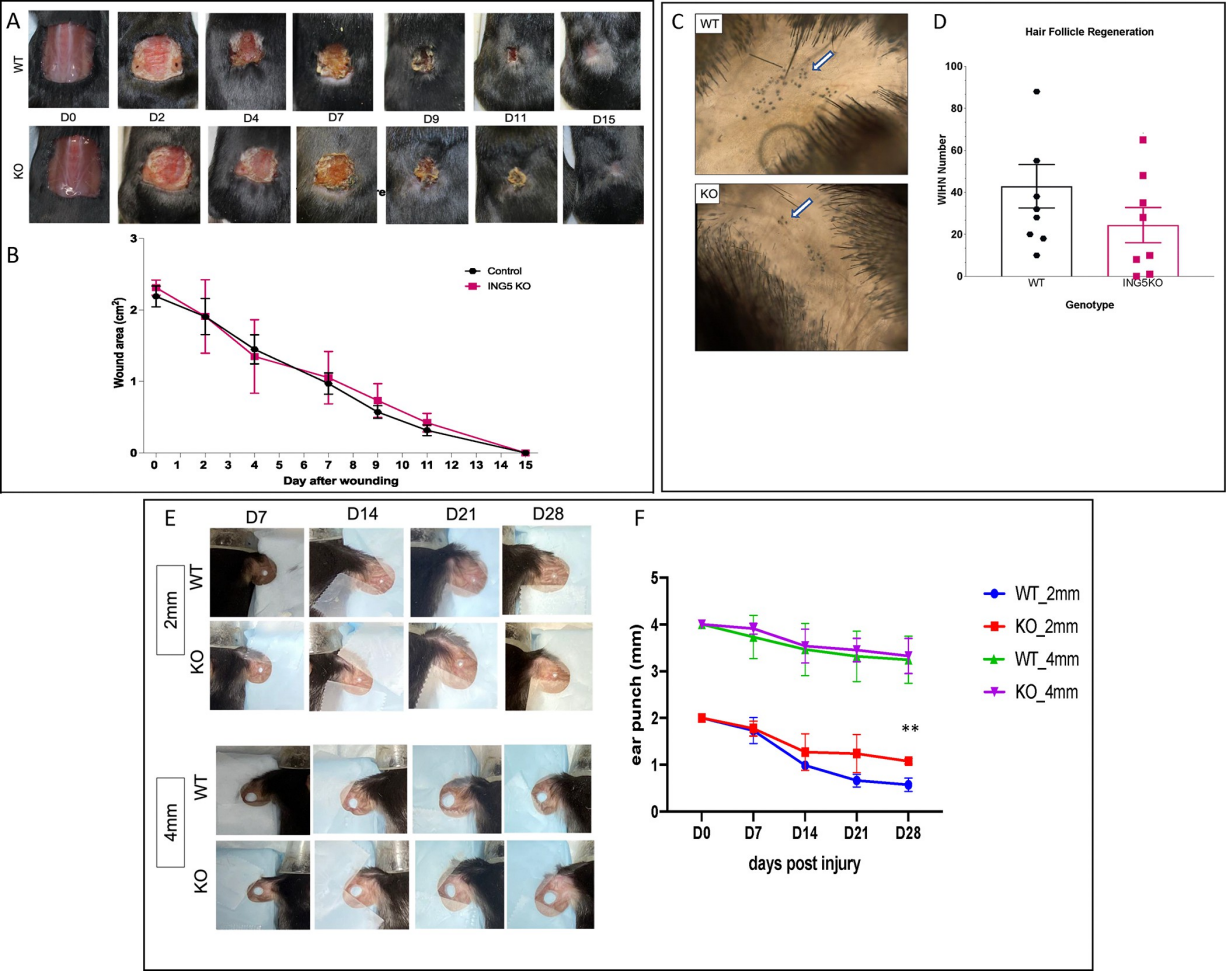
ING5 KO mice repair skin wounds efficiently but produce fewer hair follicles after wounding. **A)** Wound closure for full-thickness square wounds (≥1.5cm diameter) in 28 day old mice. Wounds were monitored every 48h until full healing. **B)** Quantification of the wound area using Image J. The graph shows mean ±SEM, (n = 8). The scale bar = 5 cm. **C)** Skin section from healed wounds showing newly generated hair follicles in WT and KO mice. The arrows identify follicles. **D)** Quantification of wound-induced hair follicle neogenesis (WIHN) in WT and KO mice (n = 8). **E)** Ear punchs (2mm and 4mm) for 8 week old WT and KO mice in the centre of the right and left ear, respectively. Wounds were monitored every 7 days for 28 days. **F)** Quantification of the ear punch area. The graph shows mean ±SEM. (n = 3). **P<0.01.

In an unbiased genome-wide screen to identify candidate epidermal stem cell regulators, ING5 was one of five genes identified that maintained the self-renewal status of epidermal stem cells, with global co-occupancy at H3K4me3 in genes actively expressed in stem cells and silenced during differentiation [23]. To examine the effect of ING5 loss on skin regeneration *in vivo*, mice received large full-thickness skin wounds at 28 days of age, as described previously [37]. The knockouts showed no difference in the rate of wound closure throughout 15 days of monitoring at which time wounds were fully healed (Fig 4A and 4B). However, wound-induced hair follicle neogenesis (WIHN) was reduced by nearly 50% in KO mice as estimated by counting hair follicles in areas of wound regrowth (Fig 4C and 4D), although this did not reach statistical significance due to inter-animal variation. Hair follicles are a major source of stem cells in the skin [38], although wound healing may depend more on extrafollicular progenitor cells [39], which loss of ING5 may promote [18]. These data indicate that growth and migration of cells during wound closure is not dependent on WT levels of stem cells, particularly in the hair follicle niche and that young ING5 KO mice regenerate fewer skin hair follicle stem cell populations after the trauma of wounding and regrowth. A mouse ear wound assay was next used to examine connective tissue regeneration. Although healing in mouse pinna cartilage is very limited in the C57BL/6 strain [40], these mice can heal 2 mm, but not 4 mm wounds. Although no difference was seen in healing larger wounds, knockouts had a significantly slower healing rate of 2 mm wounds (Fig 4E and 4F). Since a similar pattern was observed in hair follicle regeneration, this may indicate that lack of ING5 leading to a reduction of stem cells can disrupt the capacity of KO animals to heal injuries at a timely rate in different systems.

### Knockout of ING5 in mouse embryo fibroblasts results in DNA damage signaling and accumulation of polyploid cells due to incomplete mitosis

**Fig 5:**
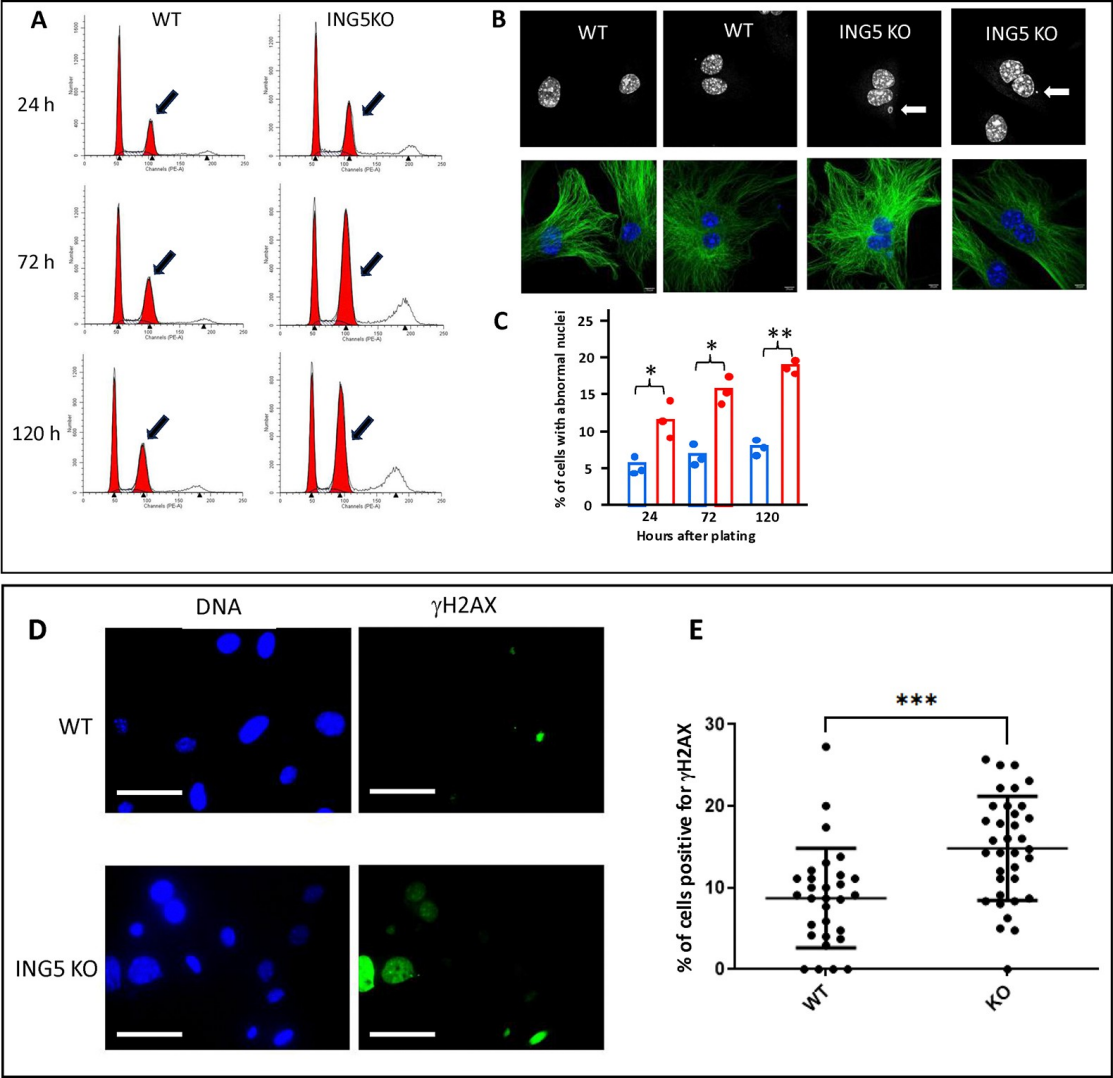
ING5 KO fibroblasts rapidly accumulate in G2 of the cell cycle. **A)** Representative flow cytometry plots for cell cycle analysis of WT and ING5-KO mouse embryo fibroblasts. Cells were harvested 24, 72 and 120 h after plating, and were harvested, fixed and stained with propidium iodide. Fifteen thousand cells were collected per sample. The arrows highlight peaks containing 4N amounts of DNA corresponding to cells containing 2 nuclei. **B)** Representative images of nuclei in WT and ING5 KO MEFs. Nuclei are stained with Hoechst and alpha-tubulin is shown in green. Arrows indicate micronuclei seen more frequently in ING5 KO cells. **C)** Quantification of the number of abnormal nuclei observed in WT and ING5 KO MEFs. Each bar graph represents 3 fields of 120 cells each counted in a blind experimental protocol. *p<0.05; **p<0.02. **D)** Representative images of WT and ING5 KO primary mouse embryo fibroblasts stained for DNA with Hoechst and with an antibody for ɣH2AX. Images were taken with a 40x objective and the bar = 50 μM. **E)** Quantification of cells positive for ɣH2AX in WT and ING5 KO primary mouse embryo fibroblasts. Cells that contained one or more foci were deemed positive. Cells with pan-nuclear staining of ɣH2AX were excluded from the analysis. 80–110 cells were counted per sample with 3 biological replicates for each cell strain (240–330 total cells per cell strain). Graph shows mean +/- SD ***p<0.005; n = 3.

To examine the effect of ING5 loss on the growth characteristics of mouse cells *in vitro* we established primary strains of WT and ING5 KO mouse embryo fibroblasts (MEFs) and studied their growth behaviour under 5% oxygen, which reduces oxidative stress that is known to inhibit murine fibroblast growth [34]. Although we saw no significant differences in growth rate, we noted a progressive increase in the number of polyploid cells by flow cytometry (Fig 5A), and a >2-fold increase in the number of MEFs with abnormal nuclei, which included multinucleated cells and cells with micronuclei, or both (Fig 5B and 5C). Consistent with the increase in phenotypically abnormal nuclei, ING5 KO MEFs also had a significantly increased proportion of cells staining for the DNA damage marker γH2AX (Fig 5D and 5E). The inability of ING5 KO cells to efficiently complete mitosis may be related to the observation that loss of the cell cycle regulated Family With Sequence Similarity 64 Member A (FAM64A) protein that positively regulates ING5 levels [41], also results in accumulation of cells in G2/M in different cell types [42].

### ING5 KO animals develop dermatitis with ageing

**Fig 6:**
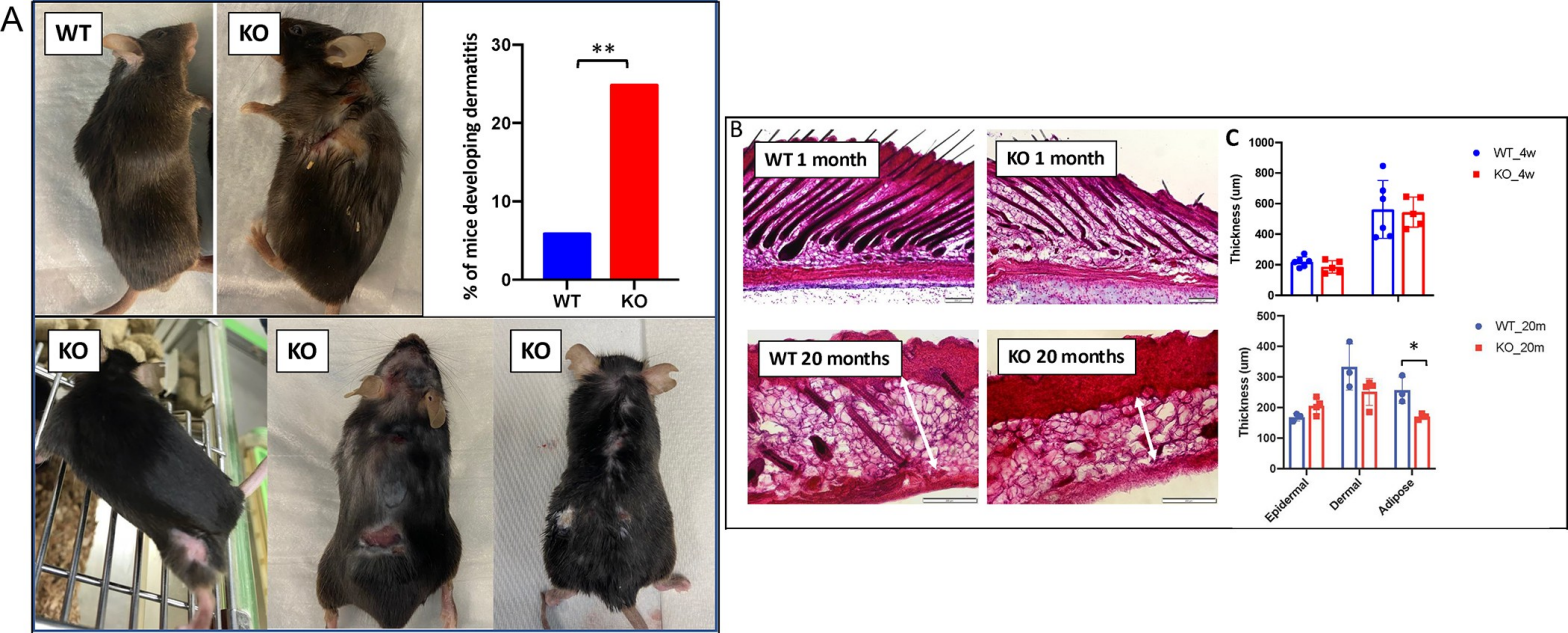
ING5 KO mice frequently develop dermatitis with ageing. **A)** WT and ING5 KO mice in upper panels are 18 months old. KO mice in lower panels show differing severities of dermatitis. The graph indicates the percentage of mice developing dermatitis by 18 months of age (n = 90 WT and 105 KO). **p<0.01. **B)** H&E staining of skin sections of WT and KO in young (1 month) and old (20 month) mice. **C)** Quantification of epidermal and dermal thickness in WT and KO young and old mice. The graph shows mean ±SEM (n = 5). Scale bar = 200 μM. *p<0.05.

With ageing, the epidermal and hair follicular stem cell reservoirs begin to decline, which affects the thickness of the epidermal and dermal layer. We noticed an ~5-fold increase in the incidence of dermatitis amongst the knockout mice that are 18 months and older when compared to their age-matched WT littermates (Fig 6A). When examining their epidermal and dermal thickness, we found no difference in thickness in 4 week old animals but the dermal layer decreased in thickness in both WT and ING5 KO animals, but to a greater degree in KO mice in animals 18–20 months old (Fig 6B and 6C). Given that cell growth rate as estimated by BrdU incorporation in murine skin was reported to not change with aging [43] but does in murine models of chronic proliferative dermatitis [44], our observations are consistent with ING5 knockout increasing the rate of differentiation in the dermis as we have noted *in vitro* [18] and in the brains of our ING5 KO mice (Fig 3). This would result in depleting mesenchymal stem cell pools. Also consistent with the dermatitis seen in these animals being a result of accelerated cell turnover from aberrant mitosis and accelerated differentiation of progenitors, they show only minor changes in cytokine expression (S2 Fig), differences in blood cell types by Complete Blood Count (CBC) (S3 Fig) and undetectable infiltration of skin by immune cells as acute models of dermatitis do [44].

### Peripheral nerve regrowth is not compromised in ING5 knockout mice

**Fig 7:**
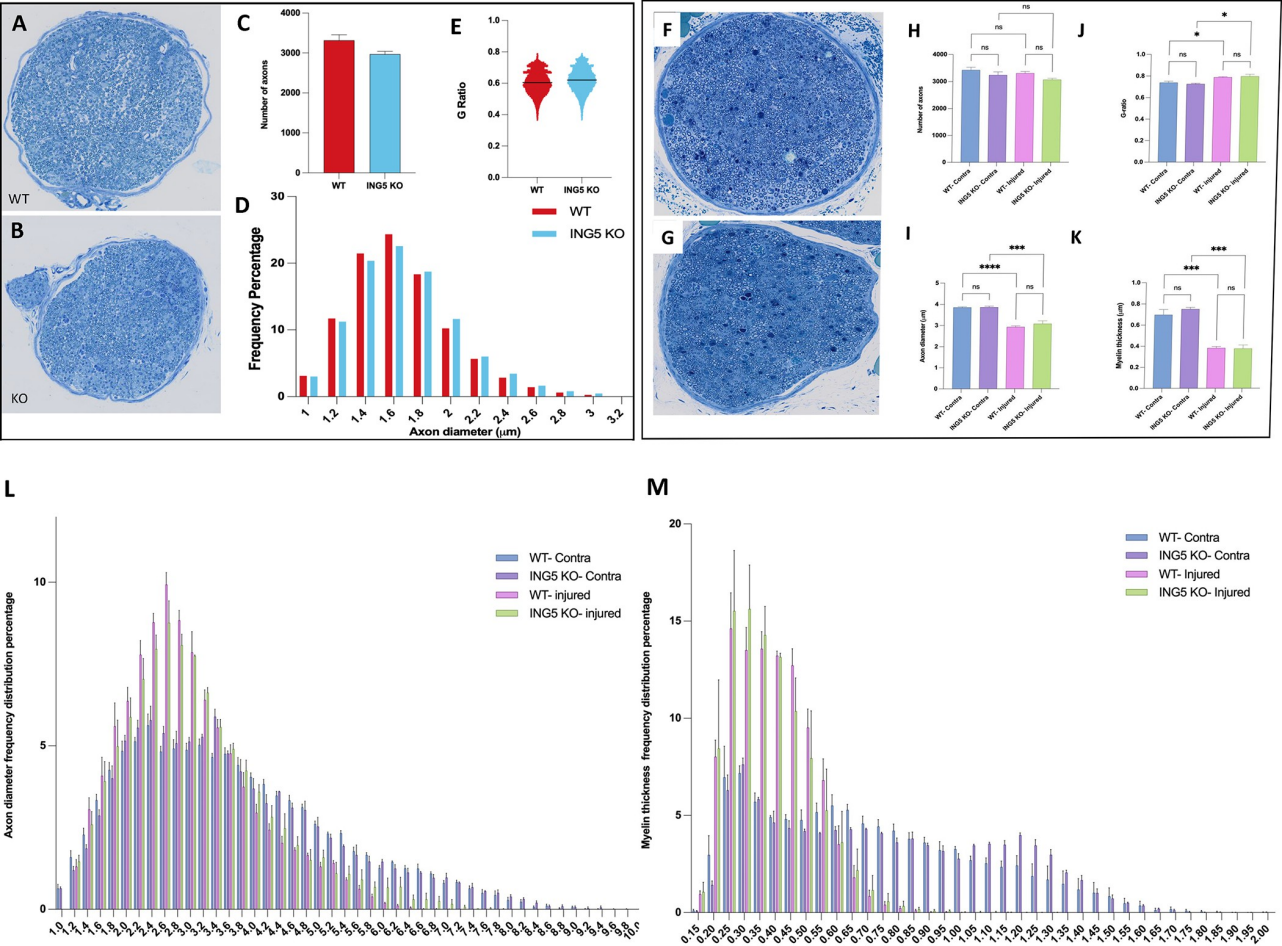
Axon regrowth and myelination is not compromised in ING5 knockouts. Neonatal wild type **A)** or knockout **B)** pups had sciatic nerves removed and semi-thin sections were stained with Toluidine blue to visualize axons and myelin. Axon number **C)**, axon diameter **D)** and G-ratio **E)** were similar in wild type and knockout mice (n = 3). Eight week-old wild type **F)** or knockout **G)** mice had sciatic nerves removed four weeks after injury and semi-thin sections were stained with toluidine blue to visualize axons and myelin. **H)** Axon number, **I)** Axon thickness**, J)** G-ratio and **K)** myelin thickness were similar in wild type and knockout mice. Axon diameter and myelin thickness was also similarly reduced in WT and ING5 KO mice after injury (n = 3). The ranges of axon diameter **L)** and myelin thickness **M)** is shown for animals in the injured and contralateral (uninjured) sciatic nerves. *P<0.05, ***<0.005.

**Fig 8:**
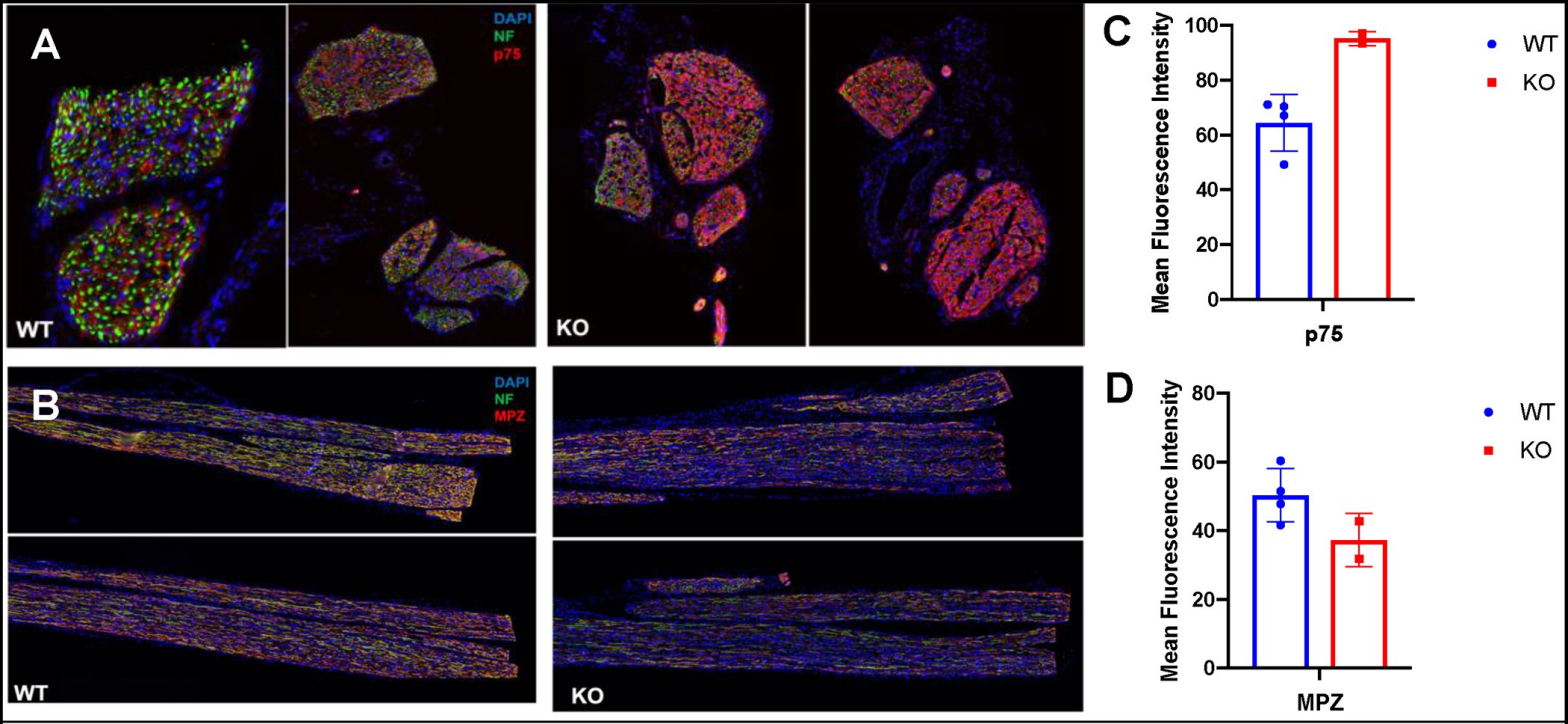
ING5 KO mice show delayed expression of the MPZ Schwann cell marker. Eight-week-old mice had their sciatic nerve crushed and harvested four weeks after injury. Harvested nerves were examined for regrowth and healing of axons by staining for the axonal neurofilament (NF) marker and for expression of MPZ marking myelinating Schwann cells or p75 marking non-myelinating Schwann cells. **A)** transverse and **B)** longitudinal sections of sciatic nerve proximal to crush points stained for the indicated markers in wild-type or ING5 KO animals. Mean fluorescence intensity for p75 **C)** and **D)** MPZ from 4 WT or 2 ING5 KO animals each, indicates that by four weeks ING5 KO animals had slightly reduced staining for markers of myelinating axons.

Examination of single cell mRNA expression data for control mice indicated that Schwann cells, which support axon regrowth expressed relatively high levels of ING5. Since injury-induced Schwann cell dedifferentiation (lineage reprogramming) is thought to contribute to nerve regeneration by multiple mechanisms including induction of cytokine and trophic factor expression [45], we asked whether Schwann cell contributions to nerve repair was compromised in the ING5 KO mice. Semi-thin transverse sections of sciatic nerves from neonatal WT and ING5 KO mice were stained with toluidine blue which provides good resolution for examining myelinated nerve fibres. Uninjured neonatal ING5 KO animals did not show significant differences in axon number, diameter or myelin thickness as expected, and the ratio of axon diameter to the diameter of axons plus myelin (G-ratio) was unchanged at 0.6, which indicates efficient nerve signal propagation which was unchanged in the knockouts (Fig 7A–7E). Adult eight-week-old animals were subjected to sciatic nerve crush procedures and four weeks later sciatic nerves were isolated and examined. As shown in (Fig 7F–7M), the numbers of axons, their diameter, myelin thickness and range of diameters were similar in WT and ING5 KO animals, with myelin thickness and axon numbers reduced similarly after injury. To ask if loss of ING5 might interfere with the ability of Schwann cells to remyelinate axons after injury, sciatic nerves from WT and ING5 KO animals were isolated four weeks after injury and examined for regeneration and the expression of markers of myelination. As seen in (Fig 8A and 8B), the extent of repair was similar in WT and ING5 KO mice as indicated by axonal neurofilament (NF) staining. Staining for the expression of p75, a marker of un-myelinating Schwann cells and for MPZ, a marker of myelinating Schwann cells (Fig 8C and 8D) indicated that the ING5 KO mice showed a reduction in the ability of Schwann cells to de-differentiate and promote axon regrowth and remyelination, although this reciprocal trend did not reach statistical significance.

### ING5 knockout mice frequently develop germinal centre diffuse large B-cell lymphoma

**Fig 9:**
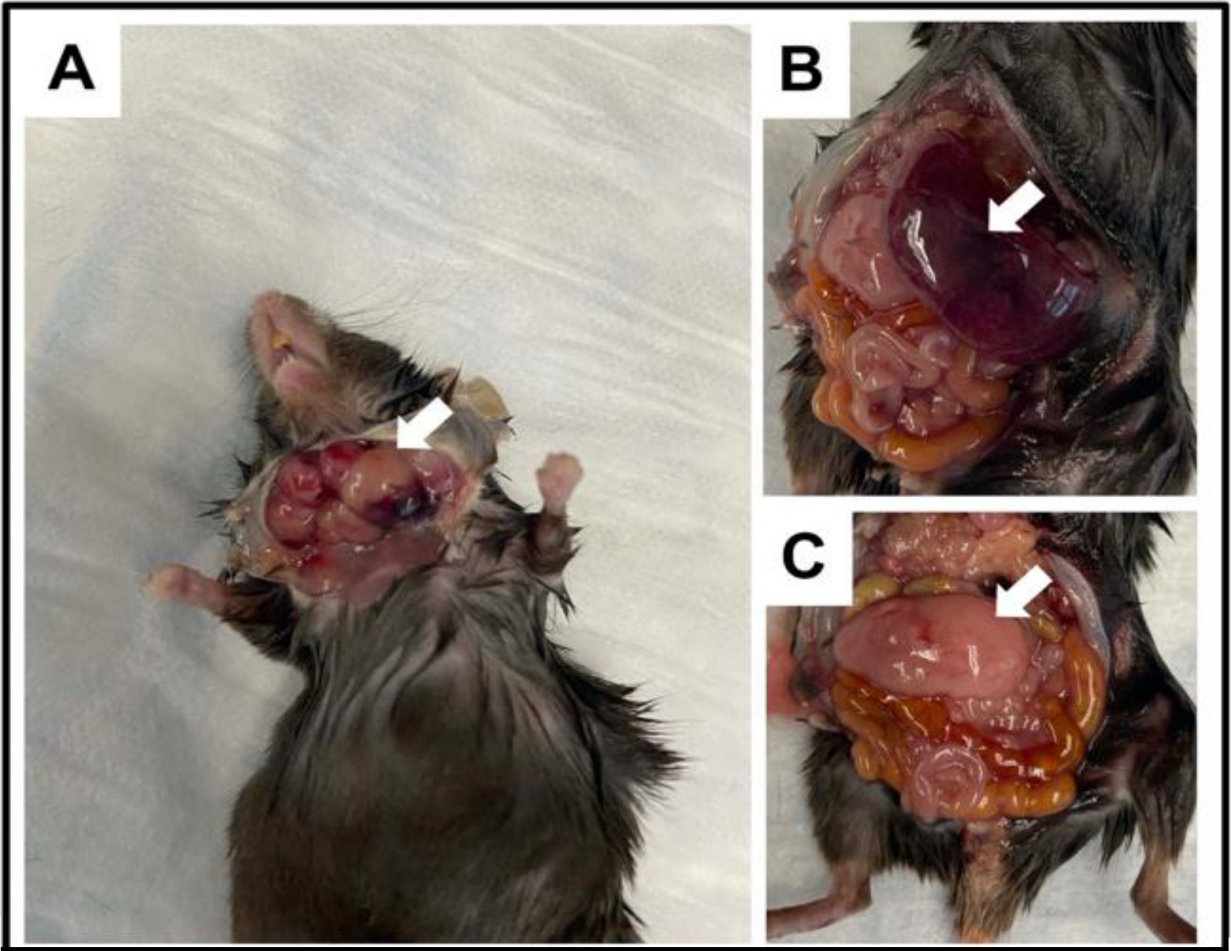
ING5 KO mice develop enlarged lymph nodes, spleens and pancreata. A subset of ING5 KO mice (17 of 129 at 18 months of age vs. 3 of 132 wild-type littermates) show enlarged lymph nodes (arrow in panel **A**), enlarged spleen (arrow in panel **B**) and enlarged pancreas (panel **C** with spleen removed). The mouse shown is 15 months old.

**Fig 10:**
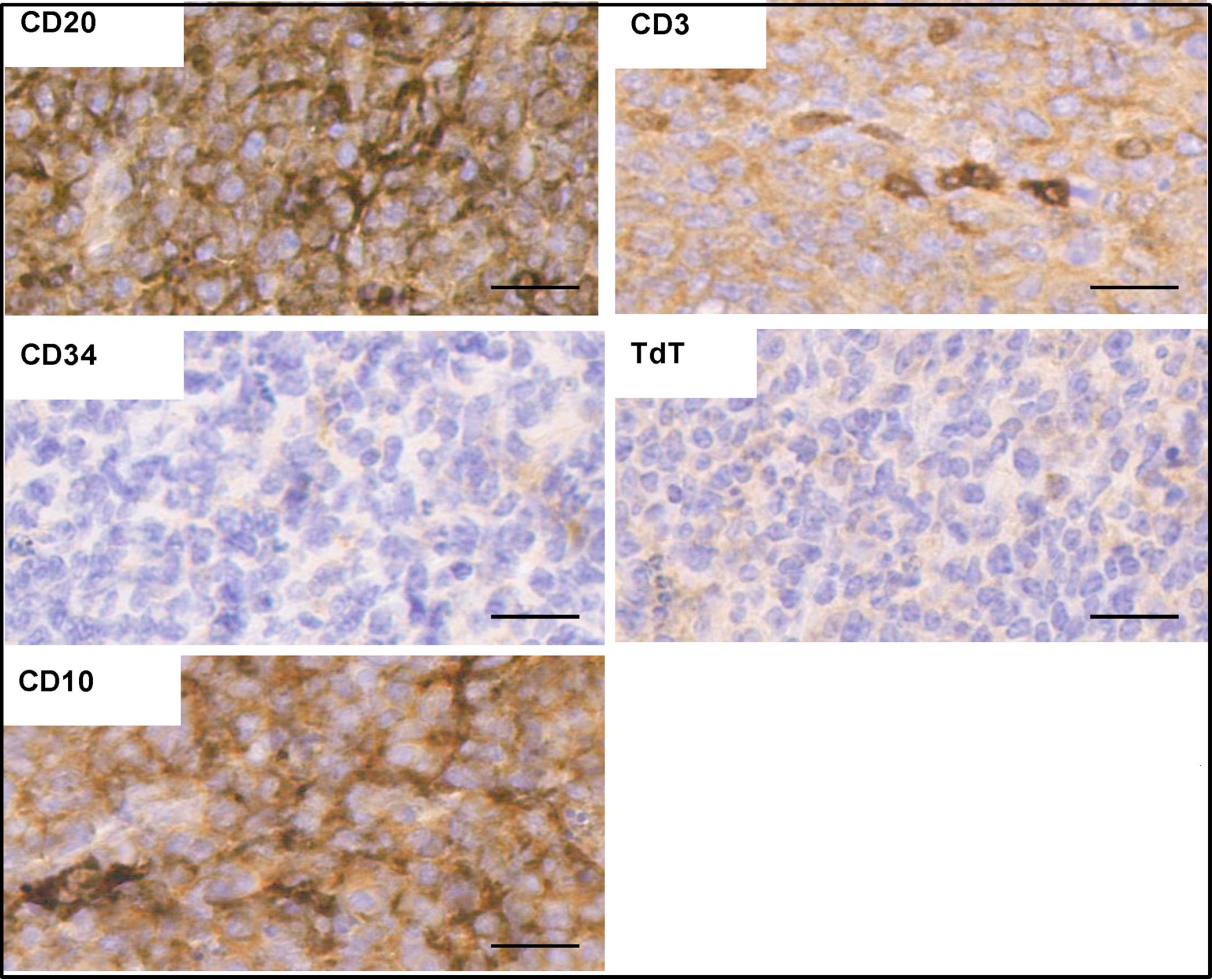
Staining ING5 KO tumors. Strong staining for CD20 and weak intermittent staining for CD3 indicate B-cell and not T-cell origin. A lack of CD34 or TdT staining indicates the tumor formed from a more mature cell lineage while CD10 staining is consistent with ING5 KO tumors representing germinal center diffuse large B-cell lymphomas. Scale bars = 50μm.

Although the majority of ING5 KO and WT animals unaffected by dermatitis had similar lifespans, living to, on average 130 weeks, 13.1% of ING5 KO animals (17 of 129) versus 2.3% of WT animals (3 of 132) that were 18 months or older developed enlarged lymph nodes, spleens and pancreata as shown in (Fig 9), and were subsequently euthanized. Localization and staining of tumours for various biomarkers (Fig 10) indicated that they are likely to be germinal centre diffuse large B-cell lymphomas (DLBCL). Staining for CD20 supports B-cell lineage while lack of CD3 staining excludes T cell origin. The lack of CD34 or TdT expression exclude a precursor lesion, and hence indicate a mature B-cell type while staining for CD10 indicates a follicular lymphoma of germinal center origin. How the ING5 KO promotes DLBCL formation is not fully understood, but the exhaustion of stem cell populations contributes to functional decline seen in advanced old age such as the inability to fight infections causing pneumonia, due to the phenomenon of immunosenescence in which cells of the immune system become unable to proliferate due to stem cell loss and cell aging/senescence. Consistent with our findings, loss of ING5 has been recently reported to reduce hematopoietic stem cell numbers [25] and single cell sequencing studies of lymphomas have implicated epigenetic regulation of immune recognition molecules in the genesis of germinal centre DLBCL [46], the form of lymphoma we see is induced by loss of the ING5 epigenetic regulator.

## Discussion

ING5 is an epigenetic regulator that binds to MOZ/MORF and HBO1 HAT complexes [12] and targets them to the H3K4Me3 mark by specific interaction with its plant homeodomain [10]. ING5 has been described as a tumor suppressor since it was both mutated and down regulated in squamous cell carcinoma [47], inhibited EMT and invasion in lung cancers [48] by targeting the WNT/b-catenin pathway [21] and regulates p53 activity by acetylation [15,49]. Consistent with affecting EMT, ING5 has also been seen to regulate stem cell differentiation in several *in vitro* systems including embryonic stem cells [18,19], epidermal stem cells [24], heart stem cells [50] and brain cancer stem (BTIC) cells [18]. To ask whether ING5 retains the ability to affect stem cell renewal and differentiation *in* vivo, we created a CRISPR/Cas9 knockout model. Although knockouts were viable with no initial observable health complications, their average litter size was 1/3 of the parental C57BL/6J mice and they had a lower rate of productive mating as previously seen for knockout of other ING family genes [29] and in particular for ING2 knockout in which sperm production is nearly eliminated [32]. Examination of young KO animals showed a doubling of differentiating neurons in the subventricular zone of the brain that harbors a high density of stem cells and a loss of stem cell marker expression, consistent with loss of ING5 promoting stem cell differentiation as seen *in vitro*. A 5-fold increase in the incidence of dermatitis was also seen in the ING5 knockouts that became obvious in middle-aged and old individuals. Given that dermatitis is frequently a result of hyperproliferation [44] and in other stem cell populations an inverse correlation between the percentage of cells undergoing DNA synthesis and stem cell numbers was reported throughout development [51], we predicted that there was likely a stem cell deficit in the skin. Skin wounding experiments confirmed that repopulation of the hair follicle stem cell niche in areas of wounding was reduced by 50% and KO animals showed a higher rate of thinning of the dermis with age, again consistent with a reduced replenishment of epidermal cells. These observations suggest a more rapid rate of stem cell differentiation and/or a lack of replenishment and subsequent expansion of rapidly growing transit amplifying/progenitor cells. Despite the observation that ING5 knockout decreases hematopoietic stem cell numbers in fetal liver, no deficiencies were reported for long-term repopulation capacity [25], suggesting that this effect was due primarily to increased stem and progenitor cell differentiation.

## Conclusions

Despite multiple changes in stem cell character in several tissues and alterations in cell cycle transit and expression of stem cell markers, we have not observed a reduction in the lifespan of ING5 KO mice that did not develop dermatitis (which necessitates euthanasia and therefore is not interpretable). Therefore, we conclude that stem cell availability for mobilization in various tissues does not limit C57BL/6J murine lifespan. This may be due to continual telomerase activity in mouse tissue, allowing stem and other cell type replenishment since even in the absence of telomere replacement in telomerase null mice, it requires 4 generations before isolated cells begin to senesce *in vitro* and 6 generations before mice lose viability [52]. This work also shows that like animals lacking ING1 [29–31], loss of ING5 results in the development of diffuse large B-cell lymphomas, confirming its status as a type II tumor suppressor.

### Animal subjects and humane endpoints

All animal procedures were performed according to our animal ethics protocol (AC22-0124), approved by the Animal Care Committee of the University of Calgary, and operating under the Guidelines of the Canadian Council on Animal Care. Experiments were run with animals from 13.5 days pc for mouse embryo fibroblast isolation to 36 months of age for lifespan determination. A total of 373 WT and 207 ING5 KO mice were used in the study with 1 wild type and 9 ING5 KO mice being found dead in their cages over a four-year period, for no reason obvious to attending veterinary staff. All others were euthanized when meeting humane endpoints or when used for specific experiments detailed in the following sections. Animals were monitored by research staff and veterinary staff twice daily. Approximately 25% of KO mice developed dermatitis and 23% of KO animals over the age of 70 months developed visible lymphomas, with both conditions resulting in activation of a humane endpoint, followed by euthanasia within 24 hours once animals showed stressed behavior. Mice exhibiting initial signs of dermatitis were housed separately, and euthanized when staff noted stressed behavior such as persistent scratching or open wounds. Experiments were performed on animals using 5% isoflurane to anesthetize while euthanasia was performed using ketamine and xylazine injection based on mouse weight, followed by cervical dislocation. Animals were also sacrificed at different time points noted in the sections below in the absence of dermatitis or lymphoma to undertake blood cell and cytokine analyses, nerve regrowth experiments and tissue analyses including morphometry and immunohistochemistry.

## Supporting information

S1 Fig Generation of CRISPR/Cas9 ING5 knockout (KO) mice. Relative ING5 mRNA levels in the brain, heart, kidney, liver, spleen, lung, and testis of ING5 KO mice compared to WT. The mRNA levels were normalized to 18S RNA. Analysis was performed for the two different mutations (1pb and 8bp deletion), using primers to amplify mRNA upstream of the mutation site (ING5_1) or downstream of the mutation site (ING5_2) as indicated by the red arrows. The graph shows mean ±SEM (n = 3).
(TIFF)

S2 Fig ING5 knockout results in minor changes in cytokine secretion. Blood was collected from WT and ING5 KO animals and analyzed using a blind experimental protocol for cytokine expression using a multiplexed array. Assays were run using 6 biological replicates each. Bars represent mean +/-SEM.
(TIFF)

S3 Fig ING5 knockout does not reproducibly affect blood cell numbers. Blood was collected from WT and ING5 KO animals and analyzed using a blind experimental protocol for complete blood count (CBC). n = 3.
(TIFF)

S1 File (DOCX)
