# Supplementary figures and images for: Knockout of the ING5 epigenetic regulator confirms roles in stem cell maintenance and tumor suppression in vivo
Source: PLoS One. 2025 Jan 9;20(1):e0313255. doi: 10.1371/journal.pone.0313255 (PMC11717183; doi:10.1371/journal.pone.0313255)

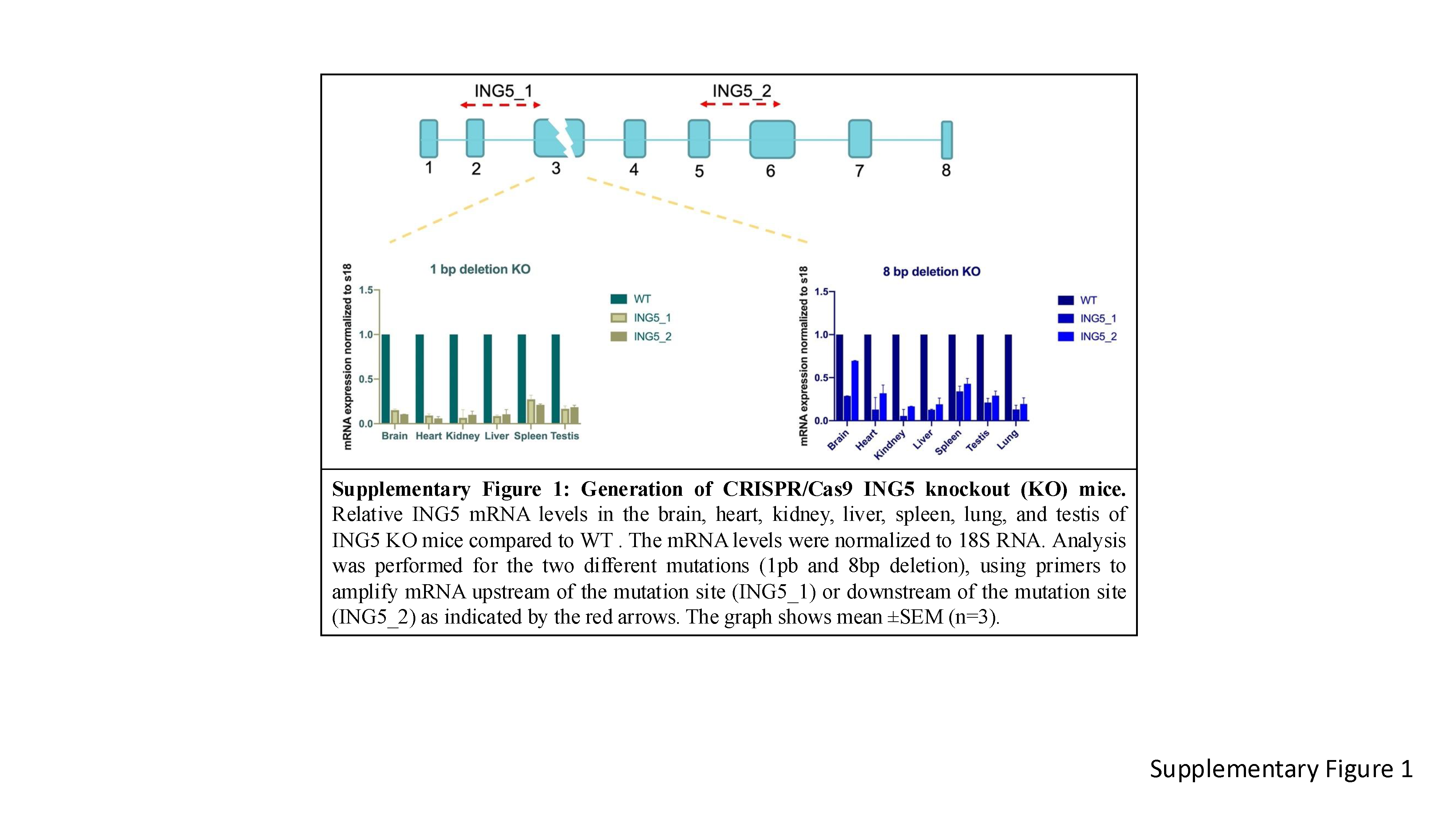

Supplement: S1 Fig — Relative ING5 mRNA levels in the brain, heart, kidney, liver, spleen, lung, and testis of ING5 KO mice compared to WT. The mRNA levels were normalized to 18S RNA. Analysis was performed for the two different mutations (1pb and 8bp deletion), using primers to amplify mRNA upstream of the mutation site (ING5_1) or downstream of the mutation site (ING5_2) as indicated by the red arrows. The graph shows mean ±SEM (n = 3). (TIFF) [file pone.0313255.s001.tiff]

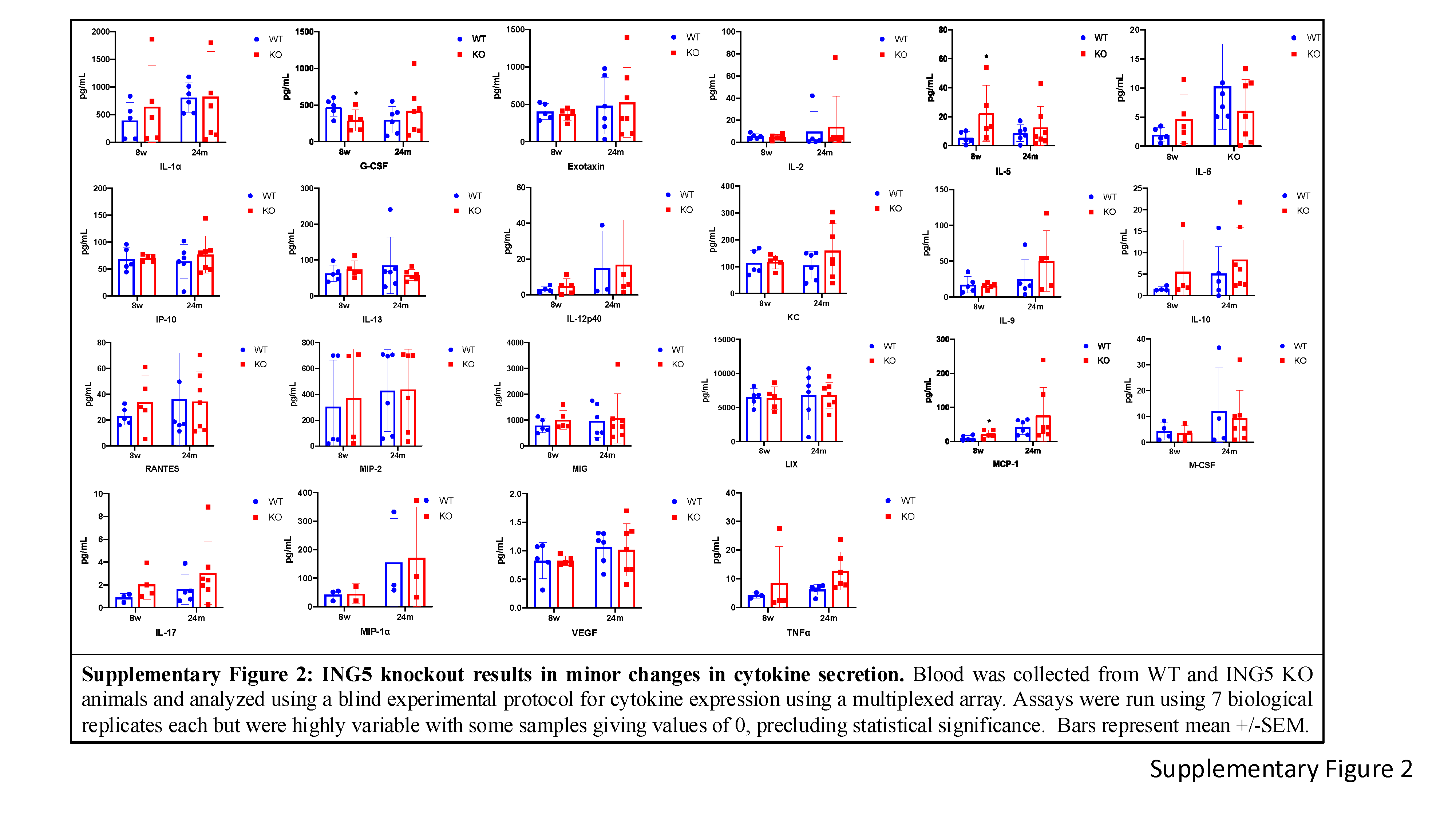

Supplement: S2 Fig — Blood was collected from WT and ING5 KO animals and analyzed using a blind experimental protocol for cytokine expression using a multiplexed array. Assays were run using 6 biological replicates each. Bars represent mean +/-SEM. (TIFF) [file pone.0313255.s002.tiff]

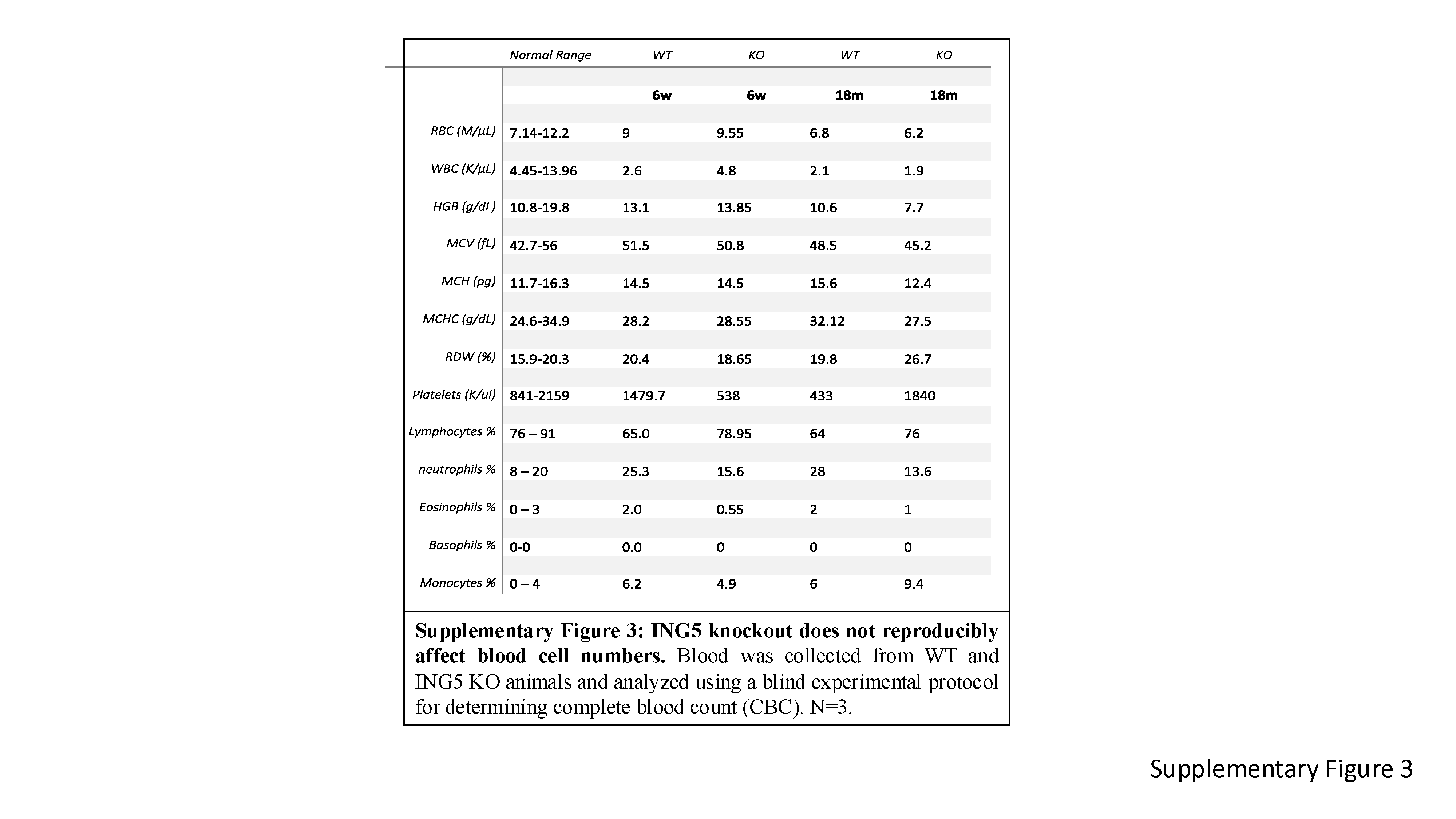

Supplement: S3 Fig — Blood was collected from WT and ING5 KO animals and analyzed using a blind experimental protocol for complete blood count (CBC). n = 3. (TIFF) [file pone.0313255.s003.tiff]
