# Supplementary material for: Knockout of the ING5 epigenetic regulator confirms roles in stem cell maintenance and tumor suppression in vivo
Source: PLoS One. 2025 Jan 9;20(1):e0313255. doi: 10.1371/journal.pone.0313255 (PMC11717183; doi:10.1371/journal.pone.0313255)
Supplement: S1 File — (DOCX) [file pone.0313255.s004.docx]

**ING5 F1 Screening: Summary (December 10, 2018)**

**PAGE Assay Results**

*Female & Male are colour-coded.*


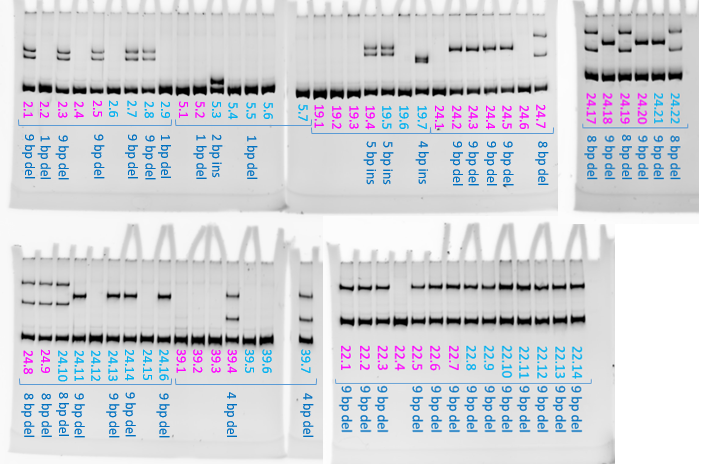


**Sequence**

**Wild-type** sequence surrounding exon 3 (UPPER CASE) is;

tgcttgtcactgcctcttacggaagtcacctgtcctcattagATAAGAAAGCAGAGATCGACATCCTGGCTGCAGAGTATATTTCCACAGTGAAGACTCTCTCGTCAGCCCAGCGTGTGGAGCACCTCCAGAAGATCCAGAGCGCCTACAGCAAGTGCAAGGAGTACAGTGATGACAAGGTGCAGCTGGCCATGCAGACCTACGAGATGgtgagtgcaggggccagtgcagcctcctgctgccttccgt

*In the mutant sequences, ~~Deleted Bases~~ & Inserted Bases are shown in colour.*

*Female & Male are colour-coded.*

**Line 2: 1 bp deletion (#2.2, #2.9) - Identical to 1 bp deletion of Line 5 -**

tgcttgtcactgcctcttacggaagtcacctgtcctcattagATAAGAAAGCAGAGATCGACATCCTGGCTGCAGAGTATATTTCCACAGTGAAGACTCTCTCGTCAGCCCAGC~~G~~TGTGGAGCACCTCCAGAAGATCCAGAGCGCCTACAGCAAGTGCAAGGAGTACAGTGATGACAAGGTGCAGCTGGCCATGCAGACCTACGAGATGgtgagtgcaggggccagtgcagcctcctgctgccttccgt

**Line 2: 9 bp deletion (#2.1, #2.3, #2.5, #2.7, #2.8)**

tgcttgtcactgcctcttacggaagtcacctgtcctcattagATAAGAAAGCAGAGATCGACATCCTGGCTGCAGAGTATATTTCCACAGTGAAGACTCTCTCGTCAGCCCAGC~~GTGTGGAGC~~ACCTCCAGAAGATCCAGAGCGCCTACAGCAAGTGCAAGGAGTACAGTGATGACAAGGTGCAGCTGGCCATGCAGACCTACGAGATGgtgagtgcaggggccagtgcagcctcctgctgccttccgt

**Line 5: 1 bp deletion (#5.2, #5.5) - Identical to 1 bp deletion of Line 2 -**

tgcttgtcactgcctcttacggaagtcacctgtcctcattagATAAGAAAGCAGAGATCGACATCCTGGCTGCAGAGTATATTTCCACAGTGAAGACTCTCTCGTCAGCCCAGC~~G~~TGTGGAGCACCTCCAGAAGATCCAGAGCGCCTACAGCAAGTGCAAGGAGTACAGTGATGACAAGGTGCAGCTGGCCATGCAGACCTACGAGATGgtgagtgcaggggccagtgcagcctcctgctgccttccgt

**Line 5: 2 bp insertion (#5.3)**

tgcttgtcactgcctcttacggaagtcacctgtcctcattagATAAGAAAGCAGAGATCGACATCCTGGCTGCAGAGTATATTTCCACAGTGAAGACTCTCTCGTCAGCCCAGCGTTTGTGGAGCACCTCCAGAAGATCCAGAGCGCCTACAGCAAGTGCAAGGAGTACAGTGATGACAAGGTGCAGCTGGCCATGCAGACCTACGAGATGgtgagtgcaggggccagtgcagcctcctgctgccttccgt

**Line 19: 4 bp insertion (#19.7)**

tgcttgtcactgcctcttacggaagtcacctgtcctcattagATAAGAAAGCAGAGATCGACATCCTGGCTGCAGAGTATATTTCCACAGTGAAGACTCTCTCGTCAGCCCAGCGTGTGGAGCACCTCCAGAATCCAGATCCAGAGCGCCTACAGCAAGTGCAAGGAGTACAGTGATGACAAGGTGCAGCTGGCCATGCAGACCTACGAGATGgtgagtgcaggggccagtgcagcctcctgctgccttccgt

**Line 19: 5 bp insertion (#19.4, #19.5)**

tgcttgtcactgcctcttacggaagtcacctgtcctcattagATAAGAAAGCAGAGATCGACATCCTGGCTGCAGAGTATATTTCCACAGTGAAGACTCTCTCGTCAGCCCAGCGTGTGGAGCACCTCCAGAATTGCAGATCCAGAGCGCCTACAGCAAGTGCAAGGAGTACAGTGATGACAAGGTGCAGCTGGCCATGCAGACCTACGAGATGgtgagtgcaggggccagtgcagcctcctgctgccttccgt

**Line 22: 9 bp deletion (#22.1, #22.2, #22.3, #22.5, #22.6, #22.7, #22.8, #22.9, #22.10, #22.11, #22.12, #22.13, #22.14) - Identical to 9 bp deletion of Line 24 -**

tgcttgtcactgcctcttacggaagtcacctgtcctcattagATAAGAAAGCAGAGATCGACATCCTGGCTGCAGAGTATATTTCCACAGTGAAGACTCTCTCGTCAGCCCAGCGTGTGGAGCACCTCCAGA**~~AGATCCAGA~~**GCGCCTACAGCAAGTGCAAGGAGTACAGTGATGACAAGGTGCAGCTGGCCATGCAGACCTACGAGATGgtgagtgcaggggccagtgcagcctcctgctgccttccgt

**Line 24: 8 bp deletion (#24.7, #24.8, #24.9, #24.10, #24.17, #24.19, #24.22)**

tgcttgtcactgcctcttacggaagtcacctgtcctcattagATAAGAAAGCAGAGATCGACATCCTGGCTGCAGAGTATATTTCCACAGTGAAGACTCTCTCGTCAGCCCAGCGTGTGGAGCACCTCCAGAA**~~GATCCAGA~~**GCGCCTACAGCAAGTGCAAGGAGTACAGTGATGACAAGGTGCAGCTGGCCATGCAGACCTACGAGATGgtgagtgcaggggccagtgcagcctcctgctgccttccgt

**Line 24: 9 bp deletion (#24.2, #24.3, #24.4, #24.5, #24.11, #24.13, #24.14, #24.16. #24.18, #24.20, #24.21) - Identical to 9 bp deletion of Line 22 -**

tgcttgtcactgcctcttacggaagtcacctgtcctcattagATAAGAAAGCAGAGATCGACATCCTGGCTGCAGAGTATATTTCCACAGTGAAGACTCTCTCGTCAGCCCAGCGTGTGGAGCACCTCCAGA**~~AGATCCAGA~~**GCGCCTACAGCAAGTGCAAGGAGTACAGTGATGACAAGGTGCAGCTGGCCATGCAGACCTACGAGATGgtgagtgcaggggccagtgcagcctcctgctgccttccgt

**Line 39: 4 bp deletion (#39.4, #39.7)**

tgcttgtcactgcctcttacggaagtcacctgtcctcattagATAAGAAAGCAGAGATCGACATCCTGGCTGCAGAGTATATTTCCACAGTGAAGACTCTCTCGTCAGCCC**~~AGCG~~**TGTGGAGCACCTCCAGAAGATCCAGAGCGCCTACAGCAAGTGCAAGGAGTACAGTGATGACAAGGTGCAGCTGGCCATGCAGACCTACGAGATGgtgagtgcaggggccagtgcagcctcctgctgccttccgt
